# Green cement production in India: prioritization and alleviation of barriers using the best–worst method

**DOI:** 10.1007/s11356-022-20217-x

**Published:** 2022-04-25

**Authors:** Marina Marinelli, Mukund Janardhanan

**Affiliations:** School of Engineering, University of Leicester, University Road, Michael Atiyah Building, Leicester, LE17RH UK

**Keywords:** BWM, Cement, Construction, Green technologies, India, Sustainability

## Abstract

Cement is a fundamental building and construction material for societies around the world. However, its manufacturing process is exceptionally energy intensive and has a substantial contribution to the man-made global warming potential which calls for immediate reduction. In this context, the implementation of green production practices and technologies in the Indian cement sector is of particular interest and global impact given that the country is the second biggest producer worldwide. Furthermore, the literature reveals that although the implementation of green practices in the cement manufacturing sector faces barriers across a variety of aspects, there is only limited research focussing on developing countries. This research covers this gap by concentrating on the barriers encountered by the cement sector of India and proposes strategies for their alleviation based on input from five experts and the use of the multi-criteria decision making method (MCDM) best–worst method (BWM). The results show that the lack of conducive corporate environment is the most important barrier, inadequate skills and attitudes are the second, while capital-related limitations come third. The experts highlight the need for mechanisms promoting cost effective environmental research and training as well as appropriate funding instruments and procurement rules from the government. Furthermore, a clear vision and plan from the management’s part are fundamental for the effective incorporation of green practices in the organisational identity and the required employee engagement.

## Introduction

Industrial revolution has started to rule the world in all aspects. As a result of the associated escalating deterioration of the environment, firms and businesses increasingly embrace environmental initiatives such as prevention of pollution, recycling, waste management, reduction of carbon emissions, and compliance to environmental legislations and regulations (Dasgupta and Das 2021). Especially the air is the most fragile environment component because it has a low capacity of neutralising pollutants that are caused by the industrial activities and can react with other chemical substances from the atmosphere, producing new substances that are dangerous for the environment and for human health (Holban et al. 2017). Given that the pollutant level of contagious gas in the atmosphere is increasing at an alarming rate (Anitha et al. 2018), several international legally binding environmental agreements have been formulated between countries in a bid to curb the increase in the harmful effects of industrial activity on nature and man (Emetere and Dania 2019). These include the Montreal Protocol on ozone protection, UN Framework convention on climate change, International Environment Protection act of 1983, and London’s clean air act of 1956. Most recently, the Paris Agreement, entered into force in late 2016, ties more than 195 nations to a commonly agreed and legally binding framework with the aim to control GHG emissions underlying climate change. The Paris agreement has the ambition to limit global temperature rise to well below 2 °C above pre-industrial levels and pursue efforts to limit the temperature increase even further to 1.5 °C (Dhar et al. 2020). Thus, it signals the end of traditional development activities based on fossil fuels and affirms that a transition to renewable energy and environmentally sustainable development is now seriously underway.

One of the most prominent trends of the recent years is the environmental performance of alternative, natural, and recycled construction materials that aim to increase the sustainability level of buildings (Kylili and Fokaides 2017). Especially with regards to cement, Orsini and Marrone (2019) confirm that the implementation of different technologies at different stages of the production process (e.g. kiln improvement, alternative fuels, recycling fines as raw materials, alternative binders, carbon capture and storage, concrete mix design, structure optimization) could be useful in enabling the cement industry to reduce its GHG emissions by 80% (compared to 1990 levels) and hit the targets set by the Paris Agreement. India’s move, however, towards a low-carbon economy, is particularly difficult as the country must balance the two goals of economic development — moving people out of poverty and providing access to electricity — and climate mitigation (Fernandes et al. 2019).

Cement is the essential “glue” in concrete, a fundamental building material for society’s infrastructure around the world. The cement manufacturing process is complex, involving multiple steps that require specialised equipment. Energy input is required at every stage, and various processes lead to emissions of CO_2_ and other greenhouse gases. On the global scale, the cement industry is responsible for 20% of the manmade CO_2_ emissions and this contributes to around 10% of the man-made global warming potential (Anand et al. 2006). Furthermore, the cement plants are one of the major contributors of air pollution. The emissions being discharged from the cement factories are responsible for the severe deterioration of the environmental quality of the factory’s surrounding area including the degradation of the air and water quality, deterioration of nearby population health, and infertility of land (Abdul Samad et al. 2020; Holban et al. 2017; Jena et al. 2020).

The Indian cement sector is the fifth-largest contributor to India’s economy and accounts for approximately 8% of global cement production, placed second worldwide (WBCSD 2019). This sector plays a fundamental role in the socio-economic growth of the nation through directly providing extremely essential support to the economy, employment and infrastructural development (Dasgupta and Das 2021). Furthermore, the India’s economy is passing through a high growth phase that is conventionally associated with the rapid rise of CO_2_-intensive infrastructure materials industries such as steel and cement (Dhar et al. 2020). Given that India’s population is set to increase by almost 40% between 2010 and 2050, the rapid urbanisation seen recently is expected to further accelerate and result in large-scale infrastructure development such as ports on the western coast, dams in the northern mountainous regions, and airports in the growing metropolitan areas (IEA 2013). The Cement Manufacturers Association (CMA) estimates that cement production in India will reach 1.360 billion tonnes annually by 2050 (WBCSD 2019). In this context and given that the industry’s operations have got it listed as a “Red Category Industry” with a pollution index of 75 by the Central Pollution Control Board (Jena et al. 2020), it is of crucial importance that green technologies and practices are implemented with the aim to reduce the harmful emissions and the respective level of atmospheric pollution. As a result, cement factories are under increasing pressure to modify their business strategies and production process (Kazancoglu et al. 2018).

However, the literature reveals that environmentally conscious initiatives and production methods very usually face barriers across a variety of aspects, which have well been documented in past research. The cement industry seems to be no exception as Anitha et al. (2018) note that controlling pollution level in atmosphere still remains a challenge for certain cement factories, while note that although the cement industry of India is amongst the most efficient in the world, it still faces many obstacles in further developing emission reduction strategies. They also note that there are only very few studies focusing on the investigation of these barriers in the context of developing countries such as India. Govindan et al. (2014) have also reported the lack of research concerning the implementation of green practices in India. Given the scarcity of the available published research as described above, as well as that the environmental performance of the cement industry of India has global impact, this research aims to cover the gap by highlighting the sustainable technologies’ implementation barriers and the strategies for their effective alleviation in the context of India. Indian experts rank these barriers with the use of best–worst method (BWM) and provide relevant suggestions.

The Research Questions (RQ) to be answered are as follows:

- RQ1: What factors are currently inhibiting the implementation of green technologies in the cement industry of India?
- RQ2: What is the relative importance of these barriers?
- RQ3: What actions need to be taken as part of an effective plan for these barriers’ alleviation?

The remainder of this paper is structured as follows. In the next section, a brief overview of related literature is provided. The subsequent section provides detailed description of the methodology applied, and “Results and discussion” provides the detailed discussion of the results obtained. “Conclusions” concludes the findings of this paper and provides future research perspectives of this work.

## Literature review

### Background

Indian cement industry has been growing at a rapid pace during the late twentieth and early twenty-first centuries; about 50% of Indian cement industry’s capacity today was built at the beginning of the 2000s. Coal is the major fuel stock for cement production in India, primarily because it is a readily available and low-cost domestic resource. There are two main sources of the gas emissions in cement manufacturing: fuel combustion used to heat raw materials and the calcination process, i.e. the decomposition of calcium carbonate to calcium oxide and CO_2_, which is responsible for around 50% of the total emissions (Govindan et al. 2014).

The study by IEA (2013) broadly highlights the following major levers for both direct and indirect reduction of emissions in the context of the Indian cement industry: the use as fuels of biomass and alternative materials like industrial or sorted municipal waste (e.g. waste tyres, sewage sludge, animal residue, waste oil, paper residue, plastic, textile), the extensive substitution of limestone by other blending materials (e.g. furnace slag or fly ash), the use of state-of-the-art technologies to increase thermal and electrical energy efficiency in cement plants, and the adaptation of waste heat recovery (WHR) technologies to convert thermal energy, otherwise lost in cement manufacture, to electricity.

Sattary and Thorpe (2016) report impressive reduction (75–90%) of carbon emitted during the manufacturing of geopolymer-based cements compared to traditional Portland cement, while Xu et al. (2013) note that the savings from reduced use of coal are in general less cost effective compared to electricity savings, mainly due to much lower coal price. Morrow et al. (2014) confirm that for India’s cement industry, the largest electricity saving potential is from low temperature WHR power generation, which saves purchased electricity by generating electricity from the waste heat onsite and replacing a ball mill with vertical roller mill in finish grinding. As per the CMA’s estimates, the WHR potential of the Indian cement sector is more than double the currently installed capacity (WBCSD 2019).

The options to mitigate CO_2_ emissions by replacing the conventional raw materials and fuels as well as substituting the conventional cement clinker are well-researched and mature technologies (Naqi and Jang 2019; Nidheesh and Kumar 2019; Salas et al. 2016) which a few years ago only enjoyed a limited degree of implementation in India (IEA 2013) but most recently this seems to be changing (Chouhan et al. 2021a, 2021b). Specifically, the alternative fuels thermal substitution rate has increased fivefold from 2010 to 2017 with the number of cement plants using them having increased from 12 plants in 2010 to 59 plants in 2016, while the sector is the largest consumer of fly ash and slag produced by India’s plants annually. The above-mentioned advancements have resulted in the sector being on track to meet its 2030 emission targets, but additional significant efforts are necessary for the additional 40% reduction required by 2050 to be achieved (WBCSD 2019). Furthermore, in an effort to address the barriers to energy efficiency within the industrial sector, the Indian Bureau of Energy Efficiency (BEE) has implemented the Performance, Achieve, and Trade (PAT) scheme that focuses on incentivizing energy efficiency in the industrial sector — including the cement sector. The selected facilities (Designated Consumers — DC) represent the highest energy consumers within each of the selected sectors and have with their own assigned target for energy consumption. This target is defined as a percentage reduction from the corresponding baseline and depends on the best performing plant within the sector on a proportional basis. BEE would issue Energy Saving Certificates (ESCerts) to DCs on achievement of the targets established, based on respective savings. DCs that do not meet the targets will be required to either buy ESCerts from entities that over achieve the targets and are issued excess certificates or pay penalties. The ESCerts will be tradable and bankable in a market between the DCs, with their price being determined by the market. In its first cycle (2012–2015), PAT has mandated an energy reduction of 6.86 million metric tons of oil equivalent (Bhandari and Shrimali 2018).

### *Barriers in green technologies implementation*

The practical side of integrating sustainability in manufacturing and the associated obstacles form a broad and well-researched area. Recent studies discussing the barriers to sustainable/green manufacturing -often in the context of SMEs or/and the automotive sector indicatively include Pathak et al. (2021), Karuppiah et al. (2020), Singh et al. (2020), Kushwaha and Talib (2020), Gohoungodji et al. (2020), Virmani et al. (2020), Malek and Desai (2020), Pathak and Singh (2019), Jaiswal and Kumar (2018), Gupta and Barua (2018), Cherrafi et al. (2017), Mittal et al. (2016), Ghazilla et al. (2015), Bhanot et al. (2017), Mittal and Sangwan (2014), and Mathiyazhagan et al. (2013). The above researches draw extensively in the literature and present similar, longer or shorter lists of barriers, an indicative representative sample of which is given in Table 1. Furthermore, barriers are also usually organised in different groups under varying headings like e.g. technological, related to knowledge and learning, economic and managerial, social and environmental, organisational and other (Malek and Desai 2020) or managerial, organisational and HR related, technological and green-resource related, financial and economic, and external stakeholders related (Gupta and Barua 2018).

**Table 1:** Representative sample of similar barriers reported in the literature

Mathiyazhagan et al. (2013)	Cherrafi et al. (2017)	Gupta and Barua (2018)	Malek and Desai (2020)	Karuppiah et al. (2020)
Lack of environmental knowledge	Lack of green thinking	Low belief in environmental benefits	Lack of awareness	Unawareness of green energy
Lack of CSR	Poor corporate culture	Lack of investment in R and D	Lack of CSR	Lack of R and D
High investment	High cost	High cost	High cost	Lack of capital
Financial constraints No bank loans Small return	Fund constraints	No bank loans	Lack of financial support, lack of funds	Lack of subsidies
Lack of communication between departments	Lack of communication and cooperation	Lack of communication	Lack of communication system	Unawareness of reputation
Lack of training	Lack of expertise training and education	Lack of training	Lack of training	Inadequate training
Lack of top management involvement	Lack of top management involvement	Lack of commitment	Unclear business case and vision	Poor organisational structure causing confusion in knowledge flow
Lack of technical expertise	Poor quality of HR	Lack of skills/expertise	Lack of technical expertise	Failure in eco-design
Inappropriate or complex technology	Lack of support for technology upgrade	Incompetent technology	Weak infrastructure	Absence of a green disposal system
Lack of performance monitoring mechanism	Unreliable data collection system	Low ability to identify opportunities	Lack of performance measurement system	Lack of recognition
Fear of failure	Fear of failure	Reluctance	Fear of failure	Lack of motivation
Feeling of no responsibility	Lack of continuous improvement culture	Fear of innovation	Lack of employee support	Lack of employee empowerment
Lack of participation in conferences/seminars	Resistance to change	Lack of government training programs	No implementation roadmap	Poor work standardization
Lack of government support	Lack of government support	Stringent unclear regulations	Lack of government policies-regulations	Lack of guidelines
Restrictive policies	Lack of organisational resources	Poor legislation enforcement	Lack of preparation	Lack of accreditation

Particularly in the context of the cement industry, an introduction to the green manufacturing concepts has been provided by Shrivastava and Shrivastava (2017), while similar barriers have been reported in the literature in the context of various developing countries like China (Hasanbeigi et al. 2013; Li et al. 2017; Liu et al. 2016, 2017a, 2017b; Wang et al. 2013; Zhang et al. 2015), Thailand (Hasanbeigi et al. 2010), Taiwan (Huang et al. 2016), Ethiopia (Tesema and Worrell 2015), Colombia (Herrera et al. 2017), and Malaysia (Goh et al. 2019). As far as India is concerned, current research in the field of cement manufacturing is very limited (Balsara et al. 2019, 2021) despite the fact that the historical coincidence of global climate change challenge with India’s high growth economic development phase poses unique threats and opportunities for aligning India’s CO_2_ emissions with the Paris agreement (Dhar et al. 2020). In this context, Dasgupta and Das (2021) very recently determined the extent of environmental management practices incorporation in 50 cement firms in India for the period 2010–2017, while only very few other publications have collected data from the sector with regard to the critical success factors or barriers, in adopting green technologies and practices. These can be found in the research reported in Seth et al. (2016) and Balsara et al. (2021), respectively.

Seth et al. (2016) identified nine general factors, i.e. the top management, the human resources management, the organisational culture, the green practices, the management of processes, and the supply chain management as critical success factors for green manufacturing in the Indian cement industry. After employing the factor analysis technique to reduce the 101 attributes involved and grouped them into 6 factors, the authors highlighted the fact that the successful implementation of green manufacturing practices demands a deeper understanding of social, organisational, and managerial factors influencing corporate performance, and goes far beyond the application of cleaner technologies and emission reduction approaches. They conclude that, among others, it requires clarity of vision, strategic direction, and commitment to green manufacturing from the part of the top management, involvement, recognition, and development culture as far as the employees are concerned, adequate funds for allocation, a corporate culture conducive to research, development and improvement, and regulatory security and compliance. These are findings similar to the ones from other industrial sectors, previously mentioned in Table 1.

Balsara et al. (2021) specifically investigated the barriers associated with applying climate change mitigation strategies in the cement industry in India. They presented a list of 26 barriers associated with economic and timeframe requirements, market restrictions, organisational and managerial inadequacies, regulatory loopholes, and technological/information gaps, i.e. a categorization similar to the one found in the literature concerning other kinds of industries. Then, they employ the fuzzy AHP (analytical hierarchy process) method and 10 expert opinions were used to prioritize the barriers. It was concluded that the group of governmental policy and regulation barriers as the main barrier based on the impact, followed by the group of organisational and managerial barriers, the group of economic and timeframe barriers, the group of technology and information barriers and lastly, and the group of market barriers. Six out of 26 barriers, all from the three first abovementioned groups, are found to be accountable for the 60% of the impact. These are the absence of financial incentives (17.99%), the uncertainty around regulations (14.06%), the investment in emission mitigation being in low priority (8.22%), the high capital requirements (7.44%), the lack of R&D facilities (6.69%), and the lack of top management commitment (6.09%). Clearly, the nature of the barriers is very similar to the ones presented in Seth et al. (2016) with the main difference being the actual ranking of the factors itself. Balsara et al.’s (2021) findings emphasize much more on the impact of externally imposed factors like legislative incentives and regulations, while Seth et al.’s (2016) analysis puts in the spotlight the development of an appropriate leadership strategy and corporate culture.

The above review of the literature reveals that the cement industry in India face similar barriers to other industrial sectors but the relative importance of the various factors needs further investigation. This research aims to add to the existing knowledge by attempting the ranking of the barriers already discussed in the literature using the BWM, a multi-criteria decision making (MCDM) technique with distinct advantages which has not been used before in the context of the sustainable development of the cement industry.

### MCDM techniques

MCDM techniques have been applied for solving a variety of problems in a vast number of areas. The related problems can be relevant to the selection of the best alternative option (e.g. Antoniou and Aretoulis 2019; Hasnain et al. 2018)) or the ranking of factors considered as drivers/barriers in order of importance/impact (e.g. Eghbali-Zarch et al. 2021; Tsz Wai et al. 2021; Zhang et al. 2021). Indicative examples from the field of sustainability in the built environment include the use of AHP for the selection of green technologies for retrofitting existing buildings (Si et al. 2016) and the use of fuzzy- AHP for the selection of sustainable materials in construction projects (Figueiredo et al. 2021). Furthermore, Asmone et al. (2019) used DEMATEL for the identification of critical indicators in the development of a green maintainability assessment system for buildings and Kamali et al. (2018) used ELECTRE and TOPSIS for the development of sustainability indices for residential modular buildings.

AHP is the method most frequently used (Mi et al. 2019); however, in cases of large number of criteria or alternatives, it involves the risk of high inconsistency and unreliable results (Malek and Desai 2020). This is because the comparisons are executed in an unstructured way (Rezaei 2015). The BWM, introduced by Rezaei (2015), alleviates this issue as compared to the AHP method, as it requires fewer pairwise comparisons and provides more consistent results using only two vectors instead of a full pairwise comparison matrix (Rezaei et al. 2016). For instance, for a MCDM problem with 10 criteria, BWM will only require 17 pairwise comparisons instead of the equivalent 45 required by AHP (Malek and Desai 2020). For all the above reasons, BWM is the method of choice in the current research. Besides, the literature confirms that despite it being recently introduced (Rezaei 2015), BWM is a very strong MCDM technique,widely used by researchers all over the world. Mi et al. (2019) provide a comprehensive bibliometric analysis of the method’s presence in the literature noting 124 relevant publications in 3 years after its introduction. The diversity of areas of implementation is also notable. Indicatively, the method has been used for: selection of suppliers (Rezaei 2016), ranking of technology enablers in SMEs (Gupta and Barua 2016), assessment of risks in chemical plants, evaluation of benefits of eco-industrial parks (Zhao et al. 2018), barriers to energy efficiency in buildings (Gupta et al. 2017), barriers to electric vehicles adoption (Tarei et al. 2021), and for the evaluation of machine and materials’ suitability for additive manufacturing (Palanisamy et al. 2020).

In BWM, for *n* criteria, the one perceived as best (*C*_*B*_) and one perceived as worst (*C*_*W*_) are determined by the expert and then, *n* − 1 pairwise comparisons are made between each of the other criteria and the best criterion *C*_*B*_ and between each of the other criteria and the worst criterion *C*_*W*_.

The steps of this method as given in Rezaei (2015) and Rezaei (2016) are:

- Step 1. Determine a set of decision criteria.
  In this step, we consider the criteria {*c*_1_, *c*_2_,…, *c*_*n*_} that should be used to arrive at a decision.
- Step 2. Determine the best (e.g. most desirable, most important) and the worst (e.g. least desirable, least important) criteria.
- Step 3. Determine the preference of the best criterion over all the other criteria using a number between 1 and 9. The resulting best-to-others vector would be: *A*_*B*_ = (*a*_*B*1_, *a*_*B*2_,…, *a*_*Bn*_),where *a*_*Bj*_ indicates the preference of the best criterion *B* over criterion *j*.
- Step 4. Determine the preference of all the criteria over the worst criterion using a number between 1 and 9. The resulting others-to-worst vector would be *A*_*w*_ = (*a*_1*w*_, *a*_*2w*_,…, *a*_*nw*_)^*T*^, where *a*_*jW*_ indicates the preference of the criterion *j* over the worst criterion *W*.
- Step 5. Find the optimal weights (*w*_1_^*^, *w*_2_^*^,…, *w*_n_^*^). The optimal weight for the criteria is the one where, for each pair of *w*_*B*_/*w*_*j*_ and *w*_*j*_/*w*_*W*_, we have *w*_*B*_/*w*_*j*_ = *a*_*Bj*_ and *w*_*j*_/*w*_*W*_ = *a*_*jW*_.

To satisfy these conditions for all *j*, we should find a solution where the maximum absolute differences *|w*_*B*_/*w*_*j*_ − *a*_*Bj*_*|* and *|w*_*j*_/*w*_*W*_ − *a*_*jW*_| for all *j* is minimized.

The equivalent problem to be solved is:

Min ξ

|*w*_*B*_/*w*_*j*_ − *a*_*Bj*_|≤ *ξ*, for all *j*

|*w*_*j*_/*w*_*W*_ − *a*_*jW*_|≤ *ξ*, for all *j*

Σ*w*_*j*_ = 1

*w*_*j*_ ≥ 0, for all *j*

Τhen, optimal weights (*w*_1_^*^, *w*_2_^*^,…, *w*_n_^*^) and ξ* can be obtained.

In order for the consistency level of the process to be checked, the consistency ratio (*CR*) can be calculated as *CR* = ξ*/*Consistency Index*, where the Consistency Index (*CI*) is a constant representing the maximum possible value of *ξ* for the given number of criteria (e.g. for 3 criteria CI = 1.00, for 5 criteria CI = 2.30, for 9 criteria CI = 5.23). The closer the *CR* is found to 0, the more consistent the comparison is. Rezaei (2016) notes that when a problem is not fully consistent (ξ* ≠ 0) then if there are more than three criteria, multiple optimal solutions may emerge. In this case, the use of a linear version of the method is preferred to allow for a unique ranking of the criteria and prevent the implications of multiple solutions.

The linear problem is the following:

Min *ξ*^*L*^

|*w*_*B*_ − *a*_*Bj*_* w*_*j*_|≤ *ξ*^*L*^, for all *j*

|*w*_*j*_ − *a*_*jW*_* w*_*W*_|≤ *ξ*^*L*^, for all *j*

Σ*w*_*j*_ = 1

*w*_*j*_ ≥ 0, for all *j*

The problem has a unique solution which comprises the optimal weights (*w*_1_^*^, *w*_2_^*^,…, *w*_n_^*^) and *ξ*^L*^ can be obtained. For this model, *ξ*^L*^ can be directly considered as an indicator of the comparisons’ consistency with values close to 0 showing a high level of consistency.

## Methodology

This research was conducted as per the following steps:

- Step 1: Thorough literature search was conducted to confirm the necessity of further investigation in the field of the cement industry of India, as established in the Introduction. The areas of green/sustainable practices and the respective challenges/issues/barriers were first investigated in the context of manufacturing in general, and following this, the search in academic databases was limited to India/the sector of cement, with very few past researches being returned, as described earlier in the literature review section.
- Step 2: An expert team of five members representing large Indian cement manufacturing companies was formed using personal contacts and online professional networking tools, in line with the purposive snowball sampling method; i.e. all the participants were selected according to their current title and experience as capable of giving the requisite information. Middle and senior engineers and managers having industrial experience of above 10 years and covering a variety of duties and specialisations were selected during the data collection process. The experts’ profiles are presented in Table 2.
- Step 3: Six categories of barriers for green/sustainable manufacturing were considered. The rationale behind the grouping was to cover both internal and external factors while keeping the groups as independent as possible so as to facilitate and simplify the process of ranking. Based on the above and taking into account the relevant groupings found in the literature, the barriers were associated with the following six aspects: governmental support, legislation, capital, personnel skills, facilities, and corporate culture. A variety of barriers previously found in the literature (Table 1) was included in each of the aforementioned categories with similar factors being summarised in clusters to facilitate the process of ranking. Eventually, the final list of barriers contains 17 factors, broken down as follows: 3 barriers related to governmental support, 2 related to legislation, 3 related to capital limitations, 3 related to personnel skills, 2 related to facilities, and 4 related to the corporate environment. The six groups of barriers were presented to the experts for them to confirm/reject these factors as barriers in the context of the cement industry of India. All the seventeen factors were confirmed by at least 3 experts and therefore, no changes occurred in the list.
- Step 4: The experts were given detailed guidance regarding the implementation steps of the BWM and the iterative application process required for the ranking of the barriers to be achieved. At first, the main barrier categories (6 groups) were ranked, and then, the sub-barriers included in each of the main categories of barriers were ranked. The BWM was applicable to the 4 out of 6 groups of barriers (governmental support, capital limitations, personnel skills and corporate environment) as these consisted of no less than 3 sub-barriers, while for the remaining two groups counting 2 sub-barriers each (facilities, legislation), the experts were asked to apply the simple additive weighting technique. The abovementioned methodological decisions were taken in line with the guidance provided by the method’s creator in their website for the method (https://bestworstmethod.com/), where the range between 3 and 9 factors is mentioned as appropriate for the method to efficiently fulfil its purpose as a ranking tool in each level of hierarchy.
- Step 5: After the ranking of the barriers was completed, the aggregated results were presented to the experts and an in depth discussion was made with each one of them with the aim to identify actions contributing to the effective mitigation of the most important barriers.

**Table 2:** Details of experts’ panel

	Expert #1	Expert #2	Expert #3	Expert #4	Expert #5
Designation	Deputy manager	Head research and development and quality assurance	Quality control Manager	Senior Manager Production department	Assistant manager
Department	Production department	Quality department	Quality department	Production department	Environment department
Years of experience in the sector	12	18	14	20	10

## Results and discussion

Table 3 presents the final list of barriers which were then ranked by the experts using the BWM. The groups were formed as explained in Step 3 above.

**Table 3:** Final list of barriers for ranking

Lack of governmental support	No financial incentives/subsidies
	No training sessions offered
	No monitoring/direction/guidelines
Environmental legislation issues	Weak enforcement
	Complex and unclear, policy gaps
Capital limitations	High initial investment
	Uncertain returns, slow payback
	No loans available
Inadequate skills and attitudes	Lack of technical expertise/knowledge
	Low environmental awareness
	Fear of failure, resistance to change
Inadequate facilities and systems	No appropriate infrastructure/facilities
	Unreliable data collection systems/measurements
Lack of conducive corporate environment	Low commitment of the top management to sustainability related initiatives
	No/low priority for CSR policies
	Unclear vision or improvement targets/unclear internal communication of plans
	No sustainability related R&D initiatives
	No sustainability related training initiatives

In the first round of the BWM implementation (ranking of the main categories of barriers; Table 4), the barriers that emerged as ‘Most Important’ (‘Best’) were the lack of conducive corporate environment (3 votes), the inadequate skills and attitudes (1 vote), and the capital limitations (1 vote). Similarly, the experts found that the ‘least important’ barriers (‘Worst’) were the environmental legislation issues (2 votes), inadequate facilities and systems (2 votes), and the lack of governmental support (1 vote).

**Table 4:** Main group weights allocated by the Experts as per the BWM

Group of Barriers	Expert 1	Expert 2	Expert 3	Expert 4	Expert 5	Group Average
Lack of governmental support	0.092	0.096	0.08	0.041 (W)	0.09	0.08
Environmental legislation issues	0.0402 (W)	0.0798	0.093	0.078	0.048 (W)	0.068
Capital limitations	0.153	0.16	0.16	0.116	0.37 (B)	0.191
Inadequate skills and attitudes	0.230	0.388 (B)	0.235	0.233	0.152	0.247
Inadequate facilities and systems	0.115	0.037 (W)	0.045 (W)	0.155	0.114	0.093
Lack of conducive corporate environment	0.371 (B)	0.240	0.392 (B)	0.377 (B)	0.227	0.321
*ξ*^L*^ (consistency check)	0.0886	0.0904	0.0783	0.0890	0.0852	

Following this, the experts implemented the steps of the method to indicate the relative importance of the sub-barriers within each group of barriers, as they perceive it, and the respective weights and averages were calculated accordingly (Table 4). The average of allocated weights brings the lack of conducive corporate environment in the first place (32.1%), the lack of skills and attitudes in the second (24.7%). and the factor of cost in the third (19.1%). Inadequate facilities and systems (9.3%), lack of governmental support (8%), and environmental legislation issues (6.8%) are the three groups of barriers which with considerably reduced impact, complete the list of barriers. The calculated values of *ξ*^L*^ being very close to 0 confirms the reliability of the rankings provided.

Following this, the experts implemented the steps of the BWM for the sub-barriers of each group of barriers and the respective weights and averages were calculated (Table 5). For groups with less than 3 factors, a simple allocation of relative weights was applied by the experts. Using the aggregated (average) values of weights in both hierarchy levels, the global weights and rankings were also obtained (Table 5). The top five sub-barriers accounting for an aggregated weight of 52.2% are in order of priority: the lack of sustainability-related R&D initiatives (14.09%), the low environmental awareness (11.34%), the high initial investment (9.16%), the lack of technical expertise/knowledge (9.02%), and the low commitment of the top management to sustainability-related initiatives and the no/low priority for CSR policies (8.58%).

**Table 5:** Final ranking of the barriers as per the BWM

Barriers	Expert 1	Expert 2	Expert 3	Expert 4	Expert 5	Within group average	Global average	Global ranking
No financial incentives/subsidies	0.2024	0.2462	0.6444 (B)	0.5417 (B)	0.5938 (B)	0.446	0.0355	**11**
No training sessions offered	0.0714 (W)	0.0769 (W)	0.1111 (W)	0.1667 (W)	0.125 (W)	0.110	0.0088	**17**
No monitoring/direction/guidelines	0.7262 (B)	0.6769 (B)	0.2444	0.2917	0.2813	0.444	0.0353	**12**
High initial investment	0.2444	0.6444 (B)	0.5625 (B)	0.6545 (B)	0.2917	0.480	0.0916	**3**
Uncertain returns, slow payback	0.6444 (B)	0.2444	0.3125	0.0909 (W)	0.5417 (B)	0.367	0.0701	**7**
No loans available	0.1111 (W)	0.1111 (W)	0.125 (W)	0.2545	0.1667 (W)	0.154	0.0294	**14**
Lack of technical expertise/knowledge	0.300	0.7027 (B)	0.5909 (B)	0.1034 (W)	0.125 (W)	0.364	0.0902	**4**
Low environmental awareness	0.600 (B)	0.1892	0.3182	0.6207 (B)	0.5625 (B)	0.458	0.1134	**2**
Fear of failure, resistance to change	0.1000 (W)	0.1081 (W)	0.0909 (W)	0.2759	0.3125	0.177	0.044	**10**
Low commitment of the top management to sustainability-related initiatives, no/low priority for CSR policies	0.2199	0.1187	0.5147 (Β)	0.3097	0.1724	0.2671	0.0858	**5**
Unclear vision or improvement targets/unclear internal communication of plans	0.0579 (W)	0.0639 (W)	0.0735 (W)	0.0531 (W)	0.1034 (W)	0.0704	0.0226	**15**
No sustainability-related research and development initiatives	0.5903 (B)	0.2968	0.2941	0.5487 (B)	0.4655	0.4391	0.1410	**1**
No sustainability related training initiatives/opportunities	0.1319	0.5205 (B)	0.1176	0.0885	0.2586	0.2235	0.0717	**6**
Weak enforcement	0.20	0.15	0.10	0.20	0.30	0.19	0.0129	**16**
Complex and unclear, policy gaps	0.80	0.85	0.90	0.80	0.70	0.81	0.0549	**9**
No appropriate infrastructure/facilities	0.30	0.35	0.40	0.40	0.40	0.37	0.0345	**13**
Unreliable data collection systems/measurements	0.70	0.65	0.60	0.60	0.60	0.63	0.0587	**8**

### Corporate culture

Amongst the factors of the most important barrier category, i.e. the lack of conducive corporate environment, the experts particularly highlighted the importance of R&D initiatives (at the top of the global barriers list) as well as the significance of high management commitment to sustainability policies and social responsibility (5th place). The importance of R&D has been noted in many previous researches, including those by Ghazilla et al. (2015) who rank it 1st amongst 64 barriers and Balsara et al. (2021) who rank it in the 5th place out of 25 barriers. Karuppiah et al. (2020) have also found that the lack of R&D is the main reason for setbacks in the adoption of green manufacturing practices and noted that its advancement may help in overcoming many other challenges. Therefore, should be prioritized in resource allocation by management. Schneider et al. (2011) note that R&D is a key factor in the cement industry for the required expertise and knowledge to be developed and to enable the industry to further reduce the environmental footprint of its operations and products. Dhar et al. (2020) make particular mention to the carbon capture and storage (CCS) technology that is still under development but has the potential to significantly contribute to the reduction in CO_2_ intensity of cement. Thus, they call for the sector to gain experience in this emerging technology. Along the same lines, the report by the World Business Council Sustainable Development (WBCSD) highlights the need for the sector to scale its R&D efforts and foster innovation in the field of alternative materials, particularly suggesting partnerships between the industries and research institutions (WBCSD 2019). Similar suggestions are made by the Working Group on Mitigation Instruments (WGMI) (WGMI 2019) emphasizing the fact that more industrial and academic R&D is required to better understand the technological and financial challenges related to decarbonisation of the industrial sector.

As far as the top management’s commitment to sustainability initiatives and CSR policies is concerned, the critical importance placed by the experts to this particular factor is in line with the findings by Digalwar et al. (2017) and Malek and Desai (2020) who consider it a key driver for implementing green technologies in India’s manufacturing industry. Noting that top management commitment provides the foundation for multiple performance improvements and success of sustainability projects. Similarly, Chiappetta Jabbour et al. (2017) argue that the commitment of senior management is vital to ensure environmental vision and corporate policy throughout the organisation and thus an absolutely critical success factor. Gedam et al. (2021) also rank the factor of top management support amongst the top critical success factors arguing that management’s stance can positively affect other success factors including organizational culture, environmental training, rewards, and incentives. Similar views highlighting the crucial role of corporate leadership and setting direction toward environmental issues and the whole organization’s sustainability performance and required culture are also mentioned in several research works (Kannan et al. 2014, Mathivathanan et al. 2018, WGMI 2019, Yusliza et al. 2019).

Regarding sustainability related training opportunities within the organisation that is also being ranked highly in the list of barriers (6th place), Schneider et al. (2011) confirm that the sustainable cement production relies on well-educated and well-trained workers. They note that highly efficient training programs are crucial for the sector to respond to the challenge of reducing energy and raw material consumption while still complying with quality, performance, and cost requirements. Along the same lines, Chiappetta Jabbour et al. (2017) note that training and learning of all collaborators is a requirement for successful implementation of tools and environmental practices and in this context, the construction of a culture that considers knowledge as a vital organisational resource should be at the core of strategic planning.

As far as the clarity of environmental targets and the efficiency of relevant communication is concerned, the experts have placed it towards the bottom of the list of barriers (15^th^ place). Nevertheless, Bhandari and Shrimali (2018) note that this is an actual PAT scheme’s weakness as each cycle’s set targets concern the cycle’s duration only, i.e. 3 years, and lacks clarity over the direction in which the collective effort needs to move beyond this period.

### Inadequate skills and attitudes

The experts’ rankings in BWM reveal that human skills and attitudes are greatly valued as sustainability transformation drivers with their lack being amongst the second most important sustainability barrier with a total weight of 24.7%. Two of the three sub-barriers of the group are found in the top five places of the global barriers list, i.e. the lack of Environmental awareness ranks 2nd and the lack of technical expertise/knowledge ranks 4th. This is line with Orsini and Marrone’s (2019) findings as well as the report by WBCSD (2019), noting that India’s cement sector is currently experiencing a significant knowledge deficit which is causing an adverse impact on its growth. According to Maheshwari et al. (2020), awareness emerges as the first step towards commitment to sustainability and is related to the sharing of information from the leader of the firm to employees to introduce them to the strategic direction of the firm. With these employees get an idea about the focus of the firm and actions they would be expected to initiate related to sustainability. Awareness requires leaders being constantly updated with the industry and clearly communicating this information to the employees so that they get to appreciate the logic for sustainability and get a sense of the interest of the sector in it. Environmental awareness is a key element for developing a conductive environment that helps organisations to successfully implement sustainability initiatives (Cherrafi et al. 2017).

Awareness directly leads to skills and knowledge enhancement; Chiappetta Jabbour et al. (2017) place training, competence for greener products and processes, and total involvement of employees amongst the critical success factors that companies need to pay particular attention to, in order to increase the chances for a successful implementation of their sustainability initiative. Wijen (2014) notes that this proposition is particularly valid in the context of developing economies, while Karuppiah et al. (2020) express their support to the recently announced initiative by the Indian government for a Green Skill Development Programme. Guimarães et al. (2018) also note that the technical capacity requirement extends to the state environmental agencies as well, as they must be able to ensure that cement companies practise waste co-processing without endangering the health of workers and populations living near the industries, while preserving environmental conditions.

Regarding the third sub-barrier in the group representing behavioural and managerial aspects such as ‘fear of failure’ and ‘resistance to change’, it is being ranked 10th in the global list which confirms previous research argument that these are limitations which need to be removed (Belhadi et al. 2017; Cherrafi et al. 2017).

### Capital limitations

There is no doubt that the scarcity of funds is always a constraint, especially in the context of a developing economy such as India. As per the experts’ responses, capital limitations represent the third most important barrier to the sustainability transformation of the Indian cement sector with a weight of 19.1% which mainly represents the experts’ concerns on the high initial investment required (3rd place in the global barrier list) and the slow payback period (7th place in the global barrier list). This is in line with findings by Balsara et al. (2021) and Orsini and Marrone (2019) who also recognise these factors as significant barriers to the effective pursue of green technologies in the cement sector. Malek and Desai (2020) and Cherrafi et al. (2017) make similar observations for the manufacturing sector in general. Despite the fact that half of India’s capacity of the energy efficient and particularly expensive WHR process was installed roughly within the past 10 years, the sector needs to make significant efforts to reach the additional 40% reduction required to meet the 2050 objectives. This requires a significant increase of the WHR potential which currently presents less than half of the sector’s potential (WBCSD 2019). Therefore, the cost still poses a significant constraint given that the WHR systems cost about USD 2.4 million per MW, while the complementary energy saving option of installing captive power plants also costs about USD 1 million per MW (IEA 2013). The sector’s concerns over capital related restrictions have been most clearly reflected in the findings by Balsara et al. (2021). Along the same lines, Bhandari and Shrimali (2018) note that investments may be restricted by the requirement for a payback period not longer than 3 years, dictated by the duration of the PAT cycle and the associated planning horizon. Similarly, WGMI (2019) notes that a clear and certain payback period could motivate the company to turn to green production technologies.

### Inadequate facilities and systems

The BWM weights given by the experts show that the lack of appropriate infrastructure/facilities in the sector is not a prominent barrier, given that this sub-barrier is ranked in the 13th place. This reflects the fact that the cement sector in India has seen substantial investments and significant technologically advanced capacity added in the past decade (WBCSD 2019) in line with the National Action Plan on Climate Change which prioritises energy efficiency initiatives, including PAT (Bhandari and Shrimali 2018). Nevertheless, there are factors which still restrict the operational side of sustainability transformation of the sector and these include the availability of efficient waste-processing and blending facilities, the availability and consistency of alternative fuels, space and layout considerations at the plant, availability of water, and scale of operation of manufacturing facilities (IEA 2013, WBCSD 2019).

The aspect of data collection systems and their reliability appears to be slightly more important as per the experts’ rankings, as it has been given the 8th place in the global barrier list. This is compatible with the findings of Cherrafi et al. (2017) who note that the appropriate implementation of visual/statistical control and performance measurement systems are indispensable for organisations to identify problems in processes, evaluate the effectiveness of action plans, and monitor progress towards the goals. In the same direction, Rijhwani (2019) argue that the required mechanisms to collect activity data need an official national platform which will enable regular documenting, archiving, updating, communicating, and sharing of data, which currently most developing countries lack.

### Lack of governmental support

Τhe lack of governmental support as considered in the context of this research incorporates the dimensions of financial support, training and provision of guidance. As per the BWM, the aspect of financial support is almost equally important to the factor of effective guidance (11th and 12th places in the list) with the training dimension marking the least important barrier ranked in the 17th and last place of the list.

There is no doubt that the dimension of financial support is of increased complexity in the case of developing economies. As Thapar et al. (2016) note, in such countries, including India, there are constraints in terms of availability of monetary resources at competitive terms due to competing demands from other sectors like education, healthcare, agriculture and infrastructure. Govindan et al. (2014) also mark the lack of loan opportunities as a considerable barrier with similar experience also being reported by Cherrafi et al. (2017).

Similarly, as far as the aspect of guidance is concerned, Cherrafi et al. (2017) also find this as a key barrier to sustainability, noting that governmental intervention, especially in terms of technical assistance, has shown its positive impact in many developed countries. WBCSD (2019) strongly support that appropriate regulations based on robust scientific analysis be devised to guide the industrial community, recognising the need for economically feasible alternatives.

Furthermore, despite the fact that the experts have ranked the need for government supported training events at the bottom of the list, WBCSD (2019) notes that the government’s support on sustainability related educational programmes has the potential to increase the availability of skilled resources and maximize the positive impact of such educational initiatives. This is also in line with IEA (2013) observation that the government can initiate appropriate awareness raising mechanisms to improve the social acceptance of using wastes as alternative fuel in cement kilns. Along the same lines, Mittal and Sangwan (2014) highlight the potential significance of governmental initiatives in the successful implementation of green manufacturing.

### Environmental legislation issues

It is widely accepted that a stable and clear legislative framework is an absolute requirement for any environmental transformation to be successful. The rankings given by the experts to the barriers of legislative complexity and inadequacy (9th place – middle of the list) and weak enforcement (16th place) reflect the success of legislative changes taking place in the recent years in India.

From the early 1990s up until today, climate equity has played an important role in shaping both India’s domestic and international climate policy discourse (Fernandes et al. 2019). Until few years ago, the green technologies had very low penetration in the Indian cement sector as this is reflected in statistics presented by IEA (2013), with the lack of uniform policy across all Indian states being among the reasons for the low uptake reported. The situation changed with the introduction of the PAT scheme in 2012, which has created the right incentive structure for improvement in energy efficiency, which has led to a reduction in energy intensity of steel and cement (Dhar et al. 2020). According to Rijhwani (2019) and WBCSD (2019), the PAT scheme is an innovative policy mandate, market-based instrument, which has successfully demonstrated its capacity to spur innovation in the energy-intensive industrial sector in India. However, Ciecierska-Holmes and Jörgensen (2019) also find that India’s domestic environmental politics are marked not only by impressive strides, but also by worrying inaction. Therefore, they suggest that it is high time for government, civil society, and corporate sector to shift environmental protection higher up the political agenda and to push for more significant moves towards a socially inclusive low-carbon development. Along the same lines, Dhar et al. (2020) also propose alternative intervention points to enhance the legislative framework like the national building codes specifying waste management policies such as the use of fly ash from power plants to the cement manufacturing industry.

### Proposed action plan

The previously discussed results allow for the conceptualisation of a plan of actions towards the elimination of the barriers in the most effective way. The weights attributed through the BWM reveal that any action plan for the facilitation of the implementation of green technologies in the cement sector of India needs to address three major pillars of hindrance which together represent almost the 80% of the total barrier impact: (1) knowledge and skill gaps including lack of R&D, i.e. the barriers ranked in places 1, 2, 4, and 17 accounting for the 35.34% of the total barrier impact; (2) capital related limitations, i.e. the barriers ranked in places 3, 7, 11, and 14 accounting for another 22.66%; and (3) lack of strategic plan and vision, i.e. the barriers ranked in places 5, 10, 12, and 15 accounting for the 18.77% of the total barrier impact.

#### Alleviation of knowledge and skill gaps including lack of R&D

The discussion with the experts confirmed that the sector needs to better understand the challenges related to decarbonisation and to scale its training, R&D, and innovation efforts. In this context, training needs to encompass various aspects of operation and management of modern cement plants including the manufacturing technology, the machinery, and the input materials. To ascertain an adequate training process, the organisation should stick to regular training activities, supported by experts and appropriate studying materials. Furthermore, for the initiative to be successful, the participants’ workload should be properly adjusted so that the necessary time is allowed for. Moreover, the experts suggested that the training events should not be limited to the employees but also include the wider society, with activities specifically tailored to promoting children’s and young persons’ environmental awareness.

The experts also agreed that emerging technologies such as the carbon, capture, and storage (CCS) technology and the use of alternative materials that have the potential to significantly contribute to the reduction in CO_2_ intensity of cement require further exploration. They also highlighted the fact that the higher ranked officials need to constantly keep pace with the changes in the sector and be updated on the latest technologies and practices through active participation in scientific fora, conferences, and trade associations.

Given the high ranking that capital related barriers have also taken, a cost-efficient strategy for organisations to achieve the required research and training opportunities was discussed. As per their experiences, such a strategy would include linking company-specific skills initiatives to state and national skill development initiatives and approaching local governments for funding opportunities. Furthermore, another strategically advisable move would be the collaboration with non-profit organisations including academic institutions in joint research projects and knowledge transfer partnerships. This would allow organisations to obtain technical assistance on how to improve operations and production processes and to develop training material with low commitment of own resources.

#### Alleviation of capital limitations

The experts confirmed that financial incentives and greater certainty over the payback period would be very effective in encouraging cement manufacturers to install the green facilities required at their plants. Given the scarcity of resources for direct support, an alternative suggestion would be for the government to ensure through the relevant national procurement rules the procurement of a minimum quantity of green cement every year, for the needs of state-funded infrastructure projects. Such an initiative would be in line with international practice for e.g. the UK government’s ‘deal’ with the Construction sector, first introduced in November 2017. This binds five Governmental Departments (the Department for Transport, the Department of Health and Social Care, the Department for Education, the Ministry of Justice and the Ministry of Defence) to include at least one off-site manufacture-based option at the development stage of their projects (Government 2018). The experts agreed that such favourable government policies and the associated message of strong political commitment in sustainability can generate substantial secondary benefits with financial value, in the sector, like e.g. reduced lending risks for banks and thus greater availability of loans.

#### Effective establishment of strategic plan and vision for sustainability

When the employees perceive the sustainability activities as indispensable part of their business, they develop trust in their organization and a sense of commitment to a prestigious and trustworthy organization that further enhances the organisation’s sustainability performance (George et al. 2020). There is no doubt that this virtuous circle requires a clear vision and concerted efforts from the management’s part to create awareness on all sustainability’s dimensions and enable employees perceive sustainability as a strategic means for business growth. On discussing the strategic incorporation of sustainability in the organisational identity, the experts suggested total employees’ involvement with regular meetings for the progress of ongoing sustainability initiatives to be discussed, and enhanced interdepartmental communication of performance targets with appropriate knowledge transfer activities. They also proposed the establishment of incentives for employee participation in sustainability projects, with their outcomes being considered in career progression and salary increase decisions. The required organisational changes were also discussed with consensus being reached around the fact that appropriate upskilling of employees about new processes through training and the assignment of appropriate personnel to lead and monitor each project’s progress are of crucial importance.

Οn the management level, the integration of environmental considerations into decision making at all levels and stages is of outmost importance. For instance, cement companies ought to actively pursue the mitigation of environmental harm resulting from aggregate and cement material extraction through effective and responsible initiatives for the rehabilitation of the land used for quarrying purposes. The experts argued that such initiatives are much more targeted and efficient when related to the implementation of ISO 14001 Environmental system and an Environmental Risk Prevention Program (ERPP) in the organisation. Furthermore, the continuous promotion of the non-environmental dimensions of sustainability is equally important for the establishment of the required culture, with actions aimed at the enhancement of safety at all production stages, the prevention of accidents, and the improvement of personnel welfare. These include the safety inspection of mechanical and electrical systems in place for cement haulage, the strict adherence to safety standards for loading, and unloading operations and regular safety briefings. A health management committee can be set up to safeguard compliance and provide advice on occupational and other health related issues in the organisation. A quality certification such as ISO 9001 can also greatly enhance the social responsibility strategy being implemented as per the experts’ experience.

## Conclusions

With climate change presenting enormous challenges to the whole planet, the Paris Agreement, entered into force in late 2016, ties more than 195 nations to control GHG emissions with the aim to limit global temperature rise to well below 2 °C above pre-industrial levels and pursue efforts to limit the temperature increase even further to 1.5 °C. Especially for the manufacturing of cement, which includes processes of extremely high-energy intensity, it is of crucial importance that the leading countries of the sector, such as India, implement green technologies and practices in their production processes. Nevertheless, the relevant research in the aforementioned operational and geographical context is very limited. This research covers the gap by investigating the barriers encountered in the cement sector of India as far as the implementation of carbon- reducing technologies is concerned. Particularly, it considers an aggregated set of 17 barriers which were verified and ranked according to the BWM by industry experts. The results revealed the importance of sustainability-related R&D initiatives and the need to increase general environmental awareness in the industrial sector. Furthermore, the findings highlighted the cement sector’s concerns over high costs for green technologies implementation, the current gap of technical expertise/knowledge on such technologies, and the impact of the top management’s commitment on the success of sustainability-related initiatives and CSR policies. Therefore, the most crucial factors emerging are the organisations’ commitment to training and R&D activities, as the only way for the sector to acquire the required level of skills and environmental awareness and the alleviation of capital related limitations. Therefore, both the public and the private sector need to work towards the development of cost effective mechanisms for environmental research and training promotion, while the government needs to take initiatives for financial support and facilitation of the industry with appropriate funding instruments and procurement rules. Furthermore, a clear vision and plan from the management’s part is fundamental for the effective incorporation of green practices in the organisational identity and the required employee engagement. The present study contributes in the existing body of knowledge as one of the very few studies contemplating the implementation of green technologies in the context of a developing nation’s industrial sector with global impact, such as the cement sector of India. Furthermore, it presents the application of the BWM, a relatively new ranking tool, in the exploration of manufacturing sustainability barriers. Future research could include the development of an interpretive structure model (ISM) for the deeper understanding of the interrelations between the barriers as well as the thorough investigation of successful governmental policies and legislative measures in force in other countries with significant contribution to the global production of cement. Such research would concern both developing (e.g. China, Vietnam, Turkey, Brazil) and developed countries (e.g. USA, Japan, Germany) for better understanding and interpretation of the trade-offs emerging between the various dimensions of sustainability and the effective mapping of the respective critical success factors.

## Data Availability

All data generated or analysed during this study are included in this published article.
